# The selective cognitive benefits of long-term Tai Chi practice on executive function of students: a study on young adults

**DOI:** 10.3389/fpsyg.2025.1702253

**Published:** 2026-01-09

**Authors:** Sime Nkemeni Darrin, Hong Hao, Cheng Long, Chanthavone Duangchit, Xu Fangfang, Zhang Tiegang

**Affiliations:** Wushu Department, Wushu College of Henan University, Kaifeng, China

**Keywords:** Tai Chi, cognitive flexibility, executive functions, inhibition, working memory, mindfulness

## Abstract

**Introduction:**

This study investigates the cognitive effects of long term (years of practice) Tai Chi practice, revealing mixed outcomes for executive functions. This study equally addresses the gap in research regarding the long term effects of Tai Chi, emphasizing its influence on the executive functions of young adults.

**Methods:**

Using the BRIEFA scale and tasks like the Flanker task, More Odd Shift Task, and Nback tasks, significant differences emerged between Tai Chi students and nonstudents.

**Results:**

In the results, Tai Chi students demonstrated superior inhibitory control and working memory compared to non Tai Chi students, as illustrated by BRIEFA results and performance on the back task. However, no significant difference in cognitive flexibility was found, possibly due to automatic reflexes from repetitive routines. This lack of cognitive flexibility improvement may stem from practitioners' instinctive responses to familiar movements, restricting adaptive strategy shifts. The results obtain illustrates that long term Tai Chi practice selectively enhances specific cognitive domains, notably inhibition and working memory, while leaving cognitive flexibility unchanged.

**Discussion:**

These benefits are attributed to Tai Chi's combination of physical movement, cognitive engagement, and mindfulness through rhythmic breathing, which enhances mental clarity, attentional discipline, and distraction management. This result challenges the assumption that Tai Chi uniformly enhance the cognitive function, highlighting the need for further research into its long term effects on specific cognitive domains. By elucidating these selective enhancements, future studies can explore the mechanisms underlying these outcomes and their implications for cognitive health. Such research could inform interventions aimed at optimizing Tai Chi practices to maximize cognitive benefits across diverse populations.

## 1 Introduction

Tai Chi is an ancient Chinese martial art that integrates slow, gentle physical movements with focused breathing and mindfulness (Ji et al., 2017). It has gained considerable recognition for its holistic approach to health and wellness (Darrin et al., 2025; Fong et al., 2014). Tai Chi's therapeutic effects have been extensively documented, encompassing improvements in physical health (Hawkes et al., 2014; Nkemeni and Hong, 2024), such as balance (Cui et al., 2024), muscle strength (Yang et al., 2024), cardiovascular health (Huston and McFarlane, 2016), and flexibility (Bai et al., 2023). It also offers significant psychological benefits, including stress reduction (Zhuang et al., 2023), enhanced mood (Hawkes et al., 2014), and improved sleep quality (Yang et al., 2023). Beyond its established physical and psychological benefits, a growing body of research suggests that Tai Chi may also positively impact cognitive functions, particularly executive functions (Cetin et al., 2020; Wang and Lyu, 2024; Zhang et al., 2023; Zheng et al., 2014, 2015).

Decades of research have illuminated the immediate benefits of Tai Chi, especially concerning physical health outcomes such as improved balance, flexibility, and cardiovascular fitness (Huston and McFarlane, 2016). Beyond physical gains, several studies suggest that consistent Tai Chi practice can lead to short-term improvements in cognitive functions, particularly those related to executive functioning (Cetin et al., 2020; Wang and Lyu, 2024; Zhang et al., 2023; Zheng et al., 2014, 2015). Executive functions are a set of higher-order cognitive processes that regulate and control other cognitive abilities and behaviors (Barkley, 1997; Miyake et al., 2000). They are crucial for goal-directed behavior, problem-solving, and adapting to novel or complex situations. Key components of executive function include inhibitory control (the ability to suppress pre-potent responses), working memory (the ability to hold and manipulate information in mind), and cognitive flexibility (the ability to switch between mental sets or tasks) (Barkley, 2001; Diamond, 2013; Janeh et al., 2012).

However, there remains a critical gap in our understanding of how long-term (years of practice) Tai Chi practice affects the executive functions of young adults. This is a crucial area of inquiry, as the cognitive demands placed on young adults in academic and professional settings are substantial. Furthermore, investigating the effects of Tai Chi on a younger population allows for a clearer examination of its cognitive benefits. Previous studies in this area are limited and have often produced mixed results, with some finding positive effects (Liu et al., 2021)and others reporting no significant differences in young adult elite athletes (Wang and Lu, 2022). This discrepancy highlights the need for a more comprehensive and robust investigation.

To address this gap, our study aims to investigate the impact of long-term Tai Chi practice on executive functions in young adults. Specifically, we hypothesize that long-term Tai Chi practice will be associated with superior inhibitory control, working memory, and cognitive flexibility compared to those without Tai Chi experience. We employed a multi-faceted approach, utilizing a combination of standardized neuropsychological tests (N-back task, Flanker task, and More-Odd Shift Task) and self-report measures (BRIEF-A) to provide a comprehensive assessment of executive function. By providing a deeper understanding of the specific cognitive benefits of long-term Tai Chi practice in young adults, this research can contribute to the development of targeted cognitive interventions and promote the broader adoption of Tai Chi as a means of enhancing mental wellbeing and academic performance. To investigate these long-term effects of Tai Chi on executive function, a group of participants was chosen based on their responses to the International Physical Activity Questionnaire Short Form (IPAQ-S). They completed the Behavior Rating Inventory of Executive Function–Adult Version (BRIEF-A) to assess executive function. Additionally, participants were evaluated on three core aspects of executive function: inhibition, cognitive flexibility (shift), and working memory. This assessment involved tasks such as the Flanker task, the More-odd shift task, and the n-back task.

## 2 Method

The study was approved by the local ethical committee of Henan University. Written informed consent was obtained from all participants before the start of the study. Participants were informed about the purpose of the study, their right to withdraw at any time without penalty, and the confidentiality of their data. Based on the BRIEF-A and IPAQ-S, Tai Chi and non-Tai Chi participants were recruited from the Wushu and International Education departments of Henan University. To ensure a valid comparison, participants were required to be full-time students with no prior experience in Tai Chi and were matched by gender and age to control for confounding variables.

### 2.1 Measures

The BRIEF-A is a 75-item assessment designed to evaluate executive function using a three-point Likert scale, where higher scores indicate greater difficulties. It is intended for individuals with at least fourth-grade reading proficiency. The assessment generates a Global Executive Composite (GEC) score, which includes the Behavioral Regulation Index (BRI) and the Metacognition Index (MI). The BRI comprises four areas: Inhibition, Shift, Emotional Control, and Self-Monitoring, while the MI includes Initiation, Working Memory, Planning/Organizing, Task Monitoring, and Organization of Materials (Erkkilä et al., 2018; Hauser et al., 2013). The BRIEF-A shows strong internal consistency (Cronbach alpha 0.93–0.96) and high test-retest reliability (0.93–0.94).

To objectively evaluate core executive functions, three specific computer-based tasks were administered. The Flanker task was used to assess inhibitory control by requiring participants to respond to a central target stimulus while ignoring flanking distractor stimuli. The More-Odd Shift Task, consisting of three conditions, was designed to evaluate cognitive flexibility by requiring participants to alternate between different classification rules. Finally, the N-back task, with 0-back, 1-back, and 2-back conditions, was utilized to measure working memory capacity. Participants were instructed to respond as quickly and accurately as possible in all tasks.

Statistical analyses were conducted using SPSS version 27 and Excel to rigorously evaluate the collected data. Independent samples *t*-tests were performed to compare mean differences between participant groups on all measures. A significance threshold of *p* < 0.05 was established to ascertain statistical significance.

## 3 Participant recruitment and demographics

A total of 28 students in the Tai Chi group and 34 students in the non-Tai Chi group participated initially. After a validity assessment, 21 Tai Chi students and 24 non-Tai Chi students were retained for analysis, which enhanced the robustness of the dataset for subsequent evaluations, as shown in Table 1. The data was collected from October 2024 to December 2024 at the Psychology Laboratory of Henan University. A qualified psychologist administered the tests to ensure consistency and minimize potential biases. The tests were performed in a quiet room to reduce external distractions. The tests were administered in three stages: the self-report measures and the computer-based neuropsychological tasks. The self-report measures were administered first, followed by the computer-based tasks, with a short break between the two. The total time for data collection was approximately 80 min per participant. The Tai Chi students were recruited from the Wushu major, while the non-Tai Chi students were recruited from the International Education major. The Tai Chi students had an average of 2.7 years of Tai Chi experience. Their long-term practice predominantly did not emphasize on the Tai Chi style, based on self-reported typical practice frequency ranging from 3 to 5 sessions per week, each lasting approximately 60 to 90 min. Conversely, the non-Tai Chi students had no previous experience with Tai Chi or other similar mind-body practices.

**Table 1:** Demographic distribution of the participants.

**Group**	**Gender**	**Student**	**Percent**	**Mean**	**Std. Deviation**
Tai Chi (21)	Male	15	63%	1.29	0.463
	Female	6	37%		
Non Tai Chi (24)	Male	9	38%	1.63	0.495
	Female	15	72%		

An initial data collection yielded 32 Tai Chi students and 43 non-Tai Chi students. Following a validity assessment, the final cohort for analysis consisted of 21 Tai Chi students and 24 non-Tai Chi students. The average age of the Tai Chi students was 19.43 years (SD = 1.326), while that of the non-Tai Chi students was 20.67 years. The demographic distribution of participants was examined based on gender. In the Tai Chi group (*n* = 21), 63% were male (*n* = 15) and 37% were female (*n* = 6). In the non-Tai Chi group (*n* = 24), 38% were male (*n* = 9) and 62% were female (*n* = 15).

### 3.1 Physical activity assessment

The Physical activity levels were categorized using the International Physical Activity Questionnaire Short Form (IPAQ-S) (Craig et al., 2017) (IPAQ-S) was used to categorize physical activity levels as Low, Moderate, or High based on Metabolic Equivalent of Task (MET) minutes per week. Moderate activity was defined as achieving at least 600 MET·min·wk^−1^, while high activity was defined as exceeding vigorous-intensity thresholds or totaling over 3000 MET·min·wk^−1^(Craig et al., 2017). The IPAQ-S assesses activities across various domains (work, commuting, leisure) and assigns MET values of 8.0 for vigorous intensity, 4.0 for moderate intensity, and 3.3 for walking.

Analysis of the IPAQ-S results (Table 2) confirmed that the Tai Chi group engaged in high physical activity (M = 6,460 MET·min·wk^−1^), while the non-Tai Chi group was in the moderate category (M = 1,495 MET·min·wk^−1^). To maintain cohort consistency, Tai Chi group participants who did not meet the 3000 MET·min·wk^−1^ threshold were excluded. A two-sample *t*-test confirmed a significant difference in physical activity levels between the groups (*t*_(21.5)_ = 11.25, *p* < 0.001, Cohen's *d* = 3.578), indicating the Tai Chi group was significantly more physically active. The demographic distribution of the final sample is detailed in Table 2. Where M is the mean and SD is the standard deviation (Jiang et al., 2023). This confirms that the Tai Chi group engaged in significantly higher levels of physical activity.

**Table 2:** IPAQ-S result of the selected students.

**Group**	**N**	**Mean (MET·min·wk^−1^)**.	**Std. deviation (MET·min·wk^−1^)**.	***t***	***P***
Tai Chi	21	6,460	1,985	11.25	0.000^**^
Non-Tai Chi	24	1,495	414		

^*^p < 0.05.

^**^p < 0.01.

### 3.2 Executive function task analysis method

**Figure 1:**
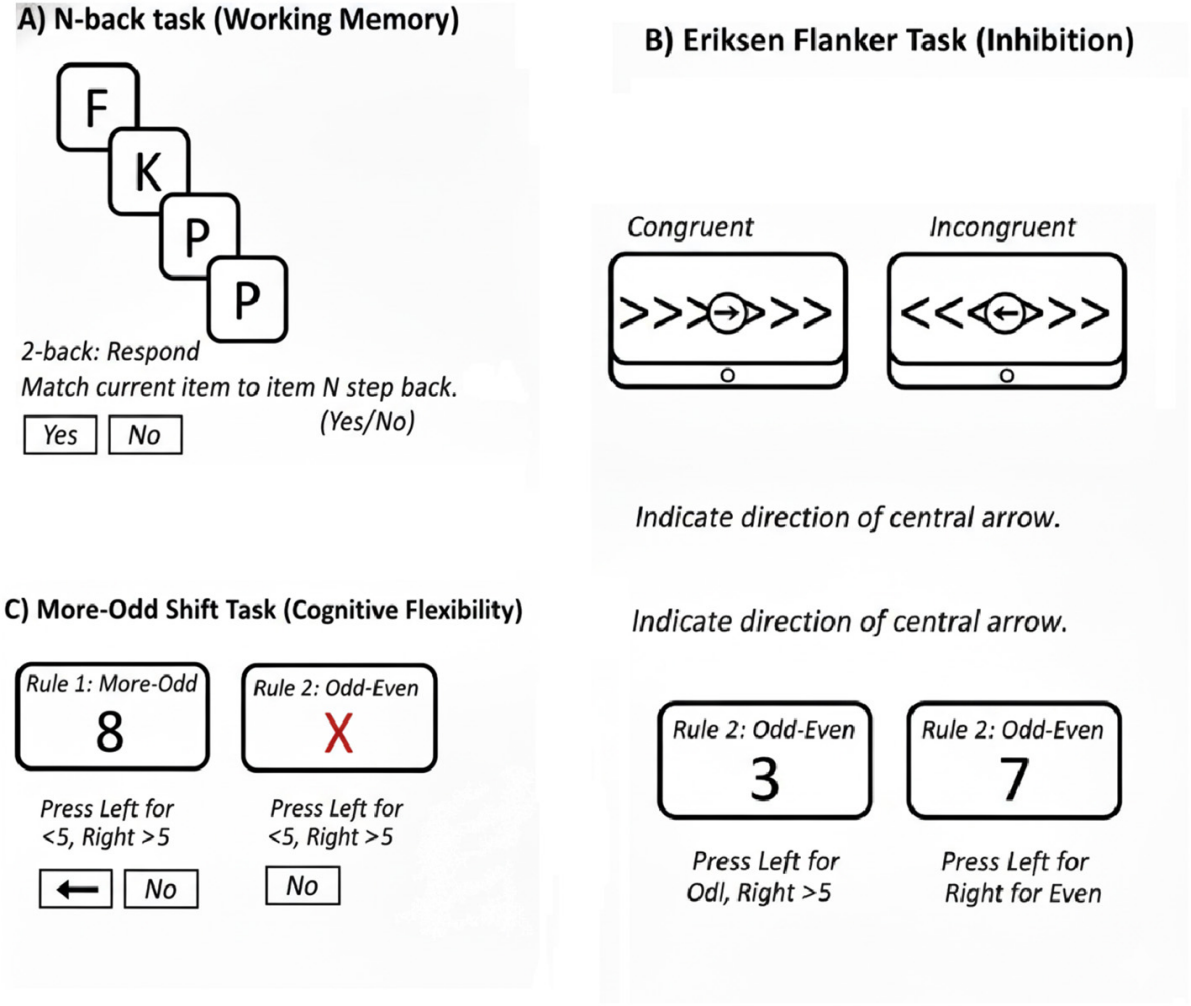
Conceptual flowchart of the experimental paradigm. This figure outlines the sequential structure of the study's cognitive assessments used to evaluate executive function components.

To provide a more objective assessment of the self-reported findings, the two groups were subjected to a battery of executive function tasks. This analysis was designed to identify differences in core cognitive abilities, focusing on inhibition, cognitive flexibility, and working memory. This investigation utilized three specific tasks: the Flanker task (Eriksen, 1995), which assesses inhibition by requiring participants to ignore distracting stimuli and respond only to relevant information; the More-Odd Shift Task (Zheng et al., 2012), designed to evaluate cognitive shifting by challenging participants to alternate between different rules; and the N-back task (Jaeggi et al., 2010), which measures working memory by requiring participants to track sequences of stimuli over several trials. Each task was strategically chosen to target distinct aspects of executive function, allowing for a comprehensive understanding of how these core factors impact the performance of each group. Through this analysis, we aim to uncover valuable insights into the nuances of executive function and how it varies between the two groups in relation to specific cognitive demands. The results of these tasks are presented below. To improve the clarity of the experimental procedure, a conceptual flowchart detailing the sequence of the cognitive assessments is provided (Figure 1).

## 4 Result

### 4.1 BRIEF-A executive function analysis

For this section, the hypotheses outlined to explore the relationship between Tai Chi and executive functions form long-term practice is: experience Tai Chi student will be associated with improved executive function performance. This hypothesis posits that individuals with advanced Tai Chi training demonstrate enhanced working memory, cognitive flexibility, and inhibitory control compared lower-level skills. The rationale behind this hypothesis is that the intricate movements and mental focus required in advanced Tai Chi practice may stimulate cognitive processes, leading to better executive functioning.

A reliability analysis was performed on the BRIEF-A scale, yielding a Cronbach's alpha coefficient of 0.971, which indicates an excellent level of internal consistency (Rabin et al., 2006; Waid-Ebbs et al., 2012) and confirms the scale is a highly reliable measure of the underlying construct of executive function (Biederman et al., 2022) as shown in Supplementary Table 3. Initial analyses examined gender differences in executive function within each group. Within the Tai Chi group, no significant gender differences were observed across any of the executive function components. This finding suggests that the effects of long-term Tai Chi practice may influence these cognitive abilities uniformly, irrespective of gender. In contrast, within the non-Tai Chi group, a significant gender difference was noted in emotional control, with males (M = 14.67, SD = 4.53) exhibiting better control than females (M = 18.67, SD = 4.05), *t*_(22)_ = −2.244, *p* = 0.035. This suggests that, in the absence of Tai Chi practice, gender differences may exist in specific executive function components, as shown in Supplementary Table 4.

A further analysis was conducted to compare the gender difference in executive function between the Tai Chi and non-Tai Chi groups, as shown in Supplementary Table 5. For females, significant disparities were noted in emotional control, with Tai Chi students (M = 13.83, SD = 2.93) exhibiting significantly lower scores than non-Tai Chi students (M = 18.67, SD = 4.05), *t*_(10)_ = −2.644, *p* = 0.016. In addition, in working memory, female Tai Chi students (M = 12.17, SD = 3.31) also scored lower than their non-Tai Chi students (M = 15.53, SD = 2.92), *t*_(19)_ = −2.299, *p* = 0.033. This indicates that Tai Chi has a positive effect in emotional regulation of female (Wang et al., 2025). For males, significant findings emerged in the Initiate measure, where Tai Chi students (M = 11.47, SD = 2.50) scored markedly lower than non-Tai Chi students (M = 14.67, SD = 2.69), *t*_(22)_ = −2.949, *p* = 0.007, indicating a significant difference in task initiation. Based on BRIEF analysis manual, lower score indicates better cognitive abilities. These results underscore the potential benefits of Tai Chi practice in enhancing emotional control, suggesting that such practice may facilitate emotional regulation and working memory capabilities, particularly for female practitioners (Wang et al., 2025).

**Figure 2:**
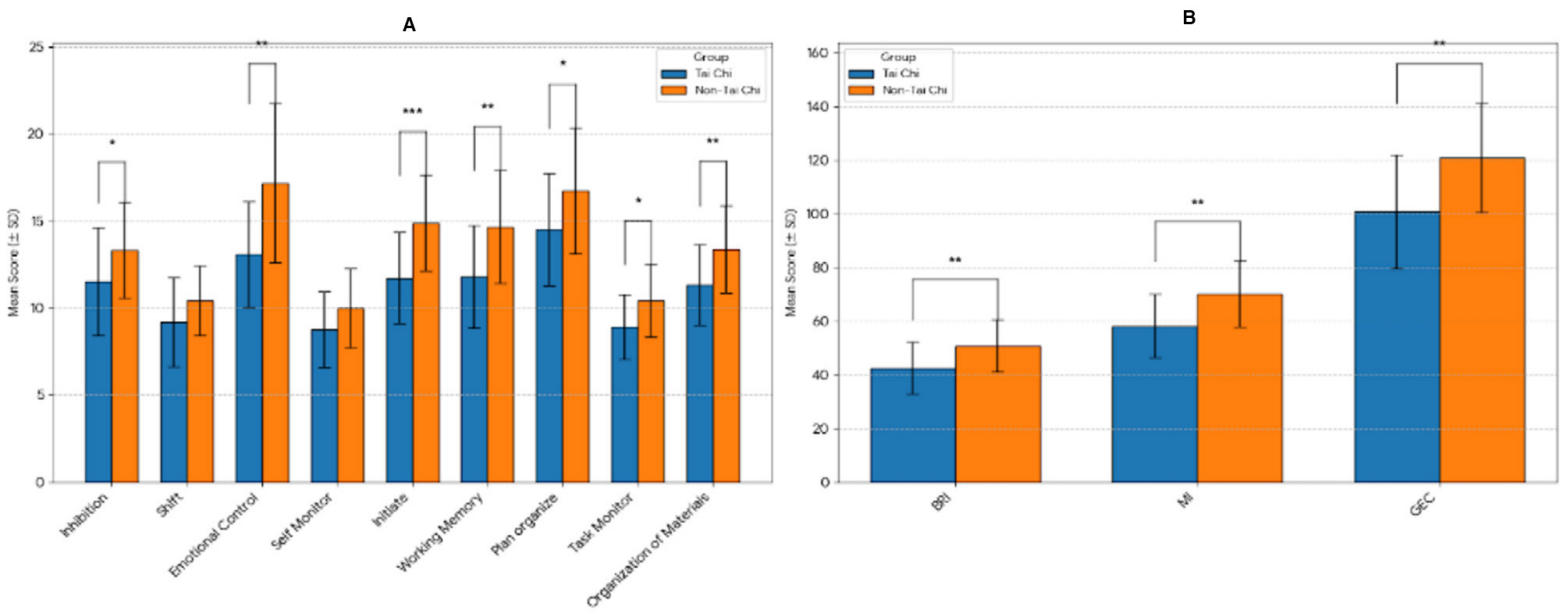
The comparison of the executive function between the two group. **(A)** Comparision of individual mean scrores. **(B)** Comparison of composite index mean scores. *p < 0.05, **p < 0.001.

Furthermore, the differences in executive function between the two groups were assessed using an independent *t*-test, as presented in Table 3 and Figure 2. This analysis highlighted distinct variations, providing insights into the impact of the Tai Chi on executive functioning.

**Table 3:** The executive function difference between the two group.

**Variable**	**Group (mean** ±**std. deviation)**		***t***	***p***	**Male (mean** ±**std. deviation)**		***t***	***p***	**Female (mean** ±**std. deviation)**		***t***	***p***
	**Tai Chi (*****n*** = **21)**	**Non-Tai Chi (*****n*** = **24)**			**Tai Chi (*****n*** = **15)**	**Non Tai Chi (*****n*** = **9)**			**Tai Chi (*****n*** = **6)**	**Non Tai Chi (*****n*** = **15)**		
Inhibition	11.52 ± 3.08	13.33 ± 2.76	−2.08	0.044^*^	11.40 ± 3.18	12.33 ± 2.69	−0.735	0.47	11.83 ± 3.06	13.93 ± 2.71	−1.548	0.138
Shift	9.19 ± 2.56	10.42 ± 2.00	−1.802	0.079	8.67 ± 2.47	9.56 ± 1.81	−0.936	0.359	10.50 ± 2.51	10.93 ± 1.98	−0.421	0.679
Emotional Control	13.10 ± 3.03	17.17 ± 4.58	−3.459	0.001^**^	12.80 ± 3.12	14.67 ± 4.53	−1.091	0.296	13.83 ± 2.93	18.67 ± 4.05	−2.644	0.016^*^
Self Monitor	8.76 ± 2.17	10.00 ± 2.27	−1.867	0.069	8.67 ± 2.13	9.78 ± 2.54	−1.153	0.261	9.00 ± 2.45	10.13 ± 2.17	−1.045	0.309
Initiate	11.71 ± 2.63	14.88 ± 2.74	−3.934	0.000^**^	11.47 ± 2.50	14.67 ± 2.69	−2.949	0.007^**^	12.33 ± 3.08	15.00 ± 2.85	−1.895	0.073
Working Memory	11.81 ± 2.94	14.67 ± 3.24	−3.079	0.004^**^	11.67 ± 2.89	13.22 ± 3.38	−1.197	0.244	12.17 ± 3.31	15.53 ± 2.92	−2.299	0.033^*^
Plan organize	14.52 ± 3.23	16.75 ± 3.60	−2.168	0.036^*^	14.27 ± 3.15	16.11 ± 4.17	−1.231	0.231	15.17 ± 3.66	17.13 ± 3.31	−1.195	0.247
Task Monitor	8.90 ± 1.84	10.42 ± 2.08	−2.563	0.014^*^	8.73 ± 1.79	10.22 ± 2.05	−1.87	0.075	9.33 ± 2.07	10.53 ± 2.17	−1.16	0.26
Organization of Materials	11.33 ± 2.31	13.38 ± 2.52	−2.821	0.007^**^	10.93 ± 2.37	12.89 ± 2.15	−2.021	0.056	12.33 ± 1.97	13.67 ± 2.74	−1.078	0.295
BRI	42.57 ± 9.75	50.92 ± 9.84	−2.85	0.007^**^	41.53 ± 9.62	46.33 ± 9.42	−1.192	0.246	45.17 ± 10.50	53.67 ± 9.31	−1.826	0.084
MI	58.29 ± 11.91	70.08 ± 12.28	−3.261	0.002^**^	57.07 ± 11.52	67.11 ± 12.22	−2.022	0.056	61.33 ± 13.41	71.87 ± 12.38	−1.722	0.101
GEC	100.86 ± 21.07	121.00 ± 20.25	−3.267	0.002^**^	98.60 ± 20.40	113.44 ± 19.29	−1.76	0.092	106.50 ± 23.60	125.53 ± 20.05	−1.872	0.077

^*^p < 0.05.

^**^p < 0.01.

Finally, a direct comparison of the two groups (Table 3) revealed that the Tai Chi group reported significantly better performance (i.e., lower scores) on multiple executive function domains. Significant differences were found for inhibition (*t*_(43)_ = −2.080, *p* = 0.044), emotional control (*t*_(43)_ = −3.459, *p* = 0.001), working memory (*t*_(43)_ = −3.079, *p* = 0.004), and overall composite scores, including the Behavioral Regulation Index (BRI) (*t*_(43)_ = −2.850, *p* = 0.007), the Metacognition Index (MI) (*t*_(43)_ = −3.261, *p* = 0.002), and the Global Executive Composite (GEC) (*t*_(43)_ = −3.267, *p* = 0.002).

No significant difference was observed in the shift component between the Tai Chi group and the non-Tai Chi group (*t*_(43)_ = −1.802, *p* = 0.079). This finding indicates that, while Tai Chi may improve many aspects of self-reported executive function, it does not appear to lead to significant improvements in the shift component. Thereby, suggests that long-term Tai Chi practice may not lead to significant improvements in cognitive flexibility.

### 4.2 Executive function task analysis

#### 4.2.1 Flankers task analysis

The Flankers task evaluates selective attention and inhibitory control. Participants respond to a central target stimulus flanked by distractor stimuli. Accurate responses depend on focusing on the target while ignoring the flanking elements (Eriksen, 1995). This task measures cognitive processing efficiency and is often used in psychological studies to understand attentional mechanisms. The analysis was conducted in two stages, examining both the accuracy of responses and the differences in reaction times between the two groups while accounting for gender and group variations. The first stage focused on assessing the correctness of responses to evaluate performance levels within each group. The second stage investigated reaction time disparities, revealing how these differences might be influenced by gender and group characteristics (Liu et al., 2022).

An independent samples *t*-test was conducted to examine differences in correct responses and reaction times on a Flanker task between Tai Chi students and non-Tai Chi students. The analysis was performed on the full sample as well as by gender

These findings suggest that female Tai Chi students do not exhibit significant differences in the incongruence reaction time to compared to non-Tai Chi students. But exhibit a difference in the proportion of incongruence correct respond which could influence the reaction time difference in a meaningful as shown in Table 4 above. It has been showed that correct response differences in the flanker task significantly affect reaction time. When participants correctly identify the target stimulus, their reaction time tends to be quicker compared to incorrect responses or when the target is flanked by incongruent stimuli. This reflects the efficiency of cognitive processes such as attention (Petersen and Posner, 2012) and inhibition, which are integral to executive function (Eriksen and Eriksen, 1974). When distractions from flankers are minimized, participants can respond faster, showcasing the task's ability to measure cognitive control in processing relevant information (Logan et al., 2024).

**Table 4:** The correct respond difference in Flanker task between the two group.

**Variable**	**Group (mean** ±**std. deviation)**		***t***	***P***	**Male (mean** ±**std. deviation)**		***t***	***p***	**Female (mean** ±**std. deviation)**		***t***	***P***
	**Tai Chi (*****n*** = **21)**	**Non-Tai Chi (*****n*** = **24)**			**Tai Chi (*****n*** = **15)**	**Non-Tai Chi (*****n*** = **9)**			**Tai Chi (*****n*** = **6)**	**Non-Tai Chi (*****n*** = **15)**		
Congruence	0.98 ± 0.02	0.98 ± 0.03	0.24	0.812	0.98 ± 0.02	0.98 ± 0.03	0.089	0.93	0.99 ± 0.01	0.98 ± 0.04	0.64	0.53
Incongruence	0.83 ± 0.11	0.82 ± 0.14	0.283	0.779	0.80 ± 0.11	0.86 ± 0.07	−1.442	0.163	0.90 ± 0.04	0.79 ± 0.16	2.344	0.031^*^

^*^p < 0.05.

^**^p < 0.01.

**Figure 3:**
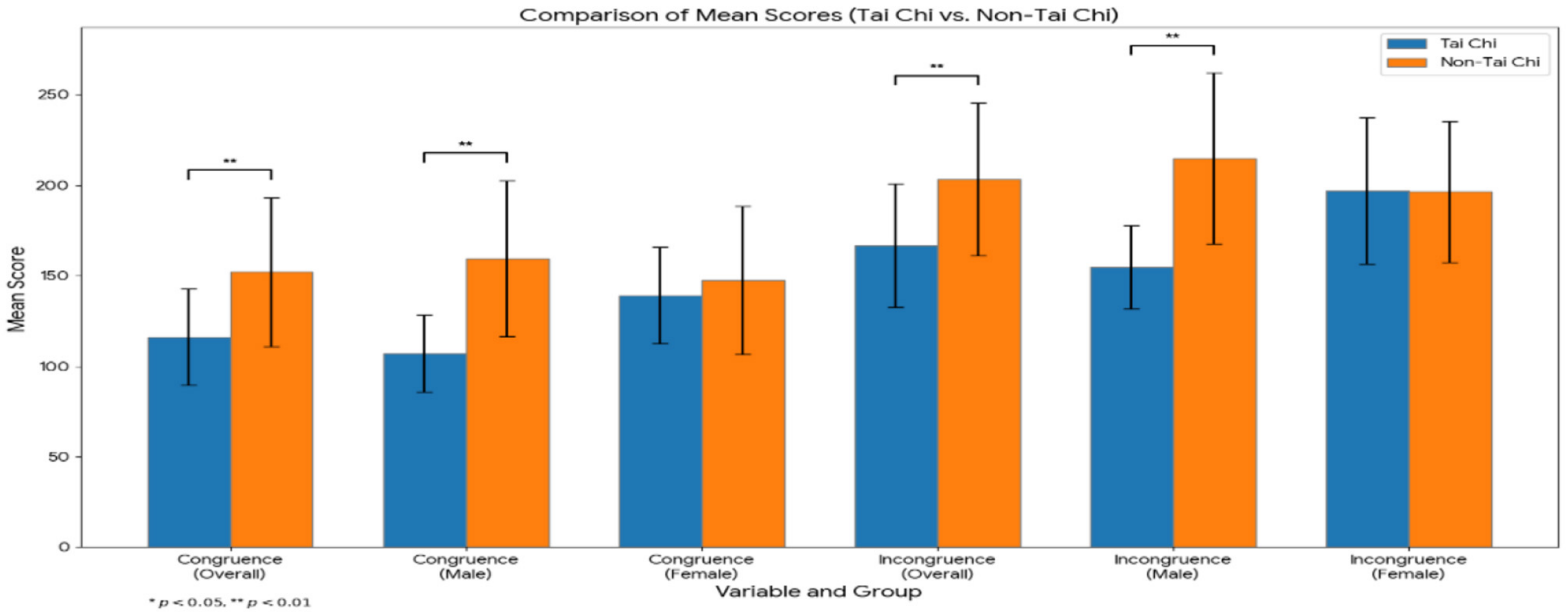
Comparison of mean reaction times (ms) on the flanker task by group (overall sample). Error bars represent ±1 standard deviation. This visualization highlights the significantly faster responses observed in the Tai Chi group for both trial types.

Additionally, the two groups were subsequently analyzed for group differences to assess the reaction time in the inhibition task. The reaction time for the Flanker's task was equally investigated through an independent samples *t*-test to compare congruence and incongruence measures between Tai Chi students and non-Tai Chi students as shown in Figure 3 and Table 5.

**Table 5:** The Flanker task reaction time responds group difference between the two group.

**Variable**	**Group (mean** ±**std. deviation)**		***t***	***p***	**Male (mean** ±**std. deviation)**		***t***	***p***	**Female (mean** ±**std. deviation)**		***t***	***p***
	**Tai Chi (*****n*** = **21)**	**Non-Tai Chi (*****n*** = **24)**			**Tai Chi (*****n*** = **15)**	**Non-Tai Chi (*****n*** = **9)**			**Tai Chi (*****n*** = **6)**	**Non-Tai Chi (*****n*** = **15)**		
Congruence	116.15 ± 26.65	151.99 ± 41.25	−3.502	0.001^**^	106.98 ± 21.14	159.45 ± 43.12	−3.413	0.006^**^	139.09 ± 26.57	147.51 ± 40.93	−0.463	0.649
Incongruence	166.73 ± 34.17	203.30 ± 42.27	−3.162	0.003^**^	154.64 ± 23.03	214.85 ± 47.29	−3.574	0.005^**^	196.94 ± 40.63	196.37 ± 39.00	0.03	0.977

^*^p < 0.05.

^**^p < 0.01.

Results indicated significant differences in both measures. For congruence, Tai Chi students (M = 116.15 ms, SD = 26.65 ms) scored significantly lower than non-Tai Chi students (M = 151.99 ms, SD = 41.25 ms), *t*_(43)_ = −3.502, *p* < 0.001. Similarly, incongruence scores were significantly lower for Tai Chi students (M = 166.73, SD = 34.17) compared to non-Tai Chi students (M = 203.30 ms, SD = 42.27 ms), *t*_(43)_ = −3.162, *p* = 0.003. This result indicates that Tai Chi group had better inhibition than non-Tai Chi group as the show lower reaction time indicating a better control in impulse.

This result was similarly observed in research pertaining to Tai Chi practice. Studies have shown that engaging in Tai Chi can enhance cognitive functions and improve attention and reaction times. For instance, a study by Wayne et al. (2014) in their systematic review demonstrated that older adults who practiced Tai Chi exhibited significantly faster reaction times compared to those who did not participate in such activities, suggesting that the mind-body exercises inherent to Tai Chi may bolster cognitive performance. Additionally, Leung et al. (2022) found that individuals who practiced Tai Chi regularly displayed enhanced executive function and better performance on tasks requiring sustained attention and quick decision-making. These findings support the notion that the meditative and physical elements of Tai Chi contribute to improved cognitive outcomes, paralleling the enhancements seen in the flanker task paradigm.

For females, no significant differences were observed in either measure. Specifically, congruence scores were similar between Tai Chi students (M = 139.09 ms, SD = 26.57 ms) and non-Tai Chi students (M = 147.51 ms, SD = 40.93 ms), *t*_(19)_ = −0.463, *p* = 0.649. Similarly, incongruence scores did not differ significantly, with Tai Chi females (M = 196.94 ms, SD = 40.63 ms) scoring similarly to non-Tai Chi females (M = 196.37 ms, SD = 39.00 ms), *t*_(19)_ = 0.03, *p* = 0.977. These findings suggest that female Tai Chi students do not exhibit significant differences in the incongruence reaction time to compared to non-Tai Chi students. But exhibit a difference in the proportion of incongruence correct respond which could influence the reaction time difference in a meaningful as shown in Table 5 above. It has been showed that correct response differences in the flanker task significantly affect reaction time. When participants correctly identify the target stimulus, their reaction time tends to be quicker compared to incorrect responses or when the target is flanked by incongruent stimuli. This reflects the efficiency of cognitive processes such as attention (Petersen and Posner, 2012) and inhibition, which are integral to executive function (Eriksen and Eriksen, 1974). When distractions from flankers are minimized, participants can respond faster, showcasing the task's ability to measure cognitive control in processing relevant information (Logan et al., 2024).

#### 4.2.2 More-odd shift task analysis

The More-Odd Shift Task serves as a cognitive assessment tool aimed at evaluating executive functions, particularly cognitive flexibility. Participants alternate responses between more and less frequent stimuli, demonstrating their adaptability to changing rules and their capacity to inhibit dominant responses. Additionally, the More-Odd Shift Task was used to examine participants' correct responses and reaction time differences to further investigate cognitive flexibility (Zheng et al., 2012).

The correct respond of the More-Odd Shift Task was investigated by independent samples *t*-test to examine gender-specific differences in performance on More1, More2, and More12 tasks between Tai Chi students and non-Tai Chi students. An independent *t*-test was employed to examine gender differences, as presented in Table 6.

**Table 6:** More-odd shift task correct responds group difference between the two group.

**Variable**	**Group (mean** ±**std. deviation)**		***t***	***p***	**Male (mean** ±**std. deviation)**		***t***	***p***	**Female (mean** ±**std. deviation)**		***t***	***p***
	**Tai Chi (*****n*** = **21)**	**Non-Tai Chi (*****n*** = **24)**			**Tai Chi (*****n*** = **15)**	**Non-Tai Chi (*****n*** = **9)**			**Tai Chi (*****n*** = **6)**	**Non-Tai Chi (*****n*** = **15)**		
More1	0.95 ± 0.11	0.96 ± 0.04	−0.529	0.6	0.93 ± 0.13	0.95 ± 0.05	−0.39	0.7	0.98 ± 0.03	0.97 ± 0.03	1.346	0.194
More2	0.95 ± 0.04	0.93 ± 0.06	0.923	0.361	0.94 ± 0.04	0.95 ± 0.03	−0.253	0.803	0.95 ± 0.04	0.92 ± 0.07	0.993	0.333
More12	0.91 ± 0.05	0.90 ± 0.08	0.364	0.718	0.91 ± 0.05	0.91 ± 0.04	−0.215	0.832	0.91 ± 0.04	0.90 ± 0.09	0.338	0.739

^*^p < 0.05.

^**^p < 0.01.

An analysis of correct responses revealed no significant performance differences between the Tai Chi and non-Tai Chi groups across any of the tasks. For the overall sample, performance on the More1 task was similar between Tai Chi students (M = 0.95, SD = 0.11) and non-Tai Chi students (M = 0.96, SD = 0.04), *t*_(43)_ = −0.529, *p* = 0.600. Similarly, no significant differences were found for the More2 task (*t*_(43)_ = 0.923, *p* = 0.361) or the More12 task (*t*_(43)_ = 0.364, *p* = 0.718). When examined by gender, this pattern persisted. Male Tai Chi students showed no significant differences from their non-Tai Chi counterparts on the More1 task (*t*_(22)_ = −0.390, *p* = 0.700), the More2 task (*t*_(22)_ = −0.253, *p* = 0.803), or the More12 task (*t*_(22)_ = −0.215, *p* = 0.832). Likewise, female Tai Chi students did not differ significantly from non-Tai Chi students on the More1 task (*t*_(19)_ = 1.35, *p* = 0.194), the More2 task (*t*_(19)_ = 0.99, *p* = 0.333), or the More12 task (*t*_(19)_ = 0.34, *p* = 0.739).

**Figure 4:**
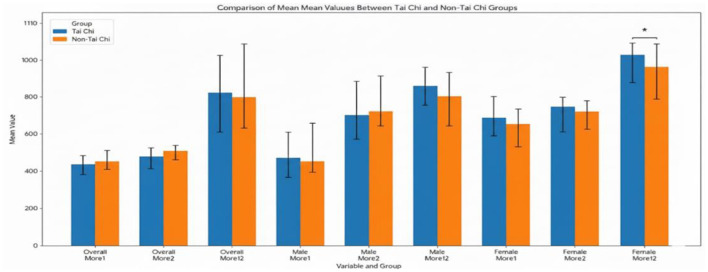
Comparison of mean reaction times (ms) on the more-odd shift task by group (overall sample). *p < 0.05, **p < 0.001.

In addition to the investigation of differences in correct responses, reaction time differences were also analyzed to assess gender and group disparities between the two groups, For the gender reaction time difference, an independent samples *t*-test was conducted to examine differences in performance on More1, More2, and More12 tasks between Tai Chi students and non-Tai Chi students as presented in Table 7 and Figure 4.

**Table 7:** More-odd shift task reactions time group difference between the two group.

**Variable**	**Group (mean** ±**std. deviation)**		***t***	***p***	**Male (mean** ±**std. deviation)**		***t***	***p***	**Female (mean** ±**std. deviation)**		***t***	***p***
	**Tai Chi (*****n*** = **21)**	**Non-Tai Chi (*****n*** = **24)**			**Tai Chi (*****n*** = **15)**	**Non-Tai Chi (*****n*** = **9)**			**Tai Chi (*****n*** = **6)**	**Non-Tai Chi (*****n*** = **15)**		
More1	570.03 ± 107.44	583.65 ± 70.17	−0.496	0.623	554.88 ± 110.11	590.14 ± 79.28	−0.836	0.412	607.89 ± 99.08	579.75 ± 66.74	0.761	0.456
More 2	616.04 ± 71.83	638.55 ± 54.75	−1.19	0.24	599.57 ± 72.35	637.31 ± 58.77	−1.322	0.2	657.21 ± 55.99	639.29 ± 54.31	0.678	0.506
More 12	866.12 ± 140.70	846.55 ± 105.13	0.533	0.597	829.96 ± 140.20	862.67 ± 134.43	−0.562	0.58	956.53 ± 102.06	836.87 ± 86.97	2.717	0.014^*^

^*^p < 0.05.

^**^p < 0.01.

More 1 = Condition for number < 5 or>5; More 2 = even or odd condition; More 12 = combined conditions of More 1 and More 2.

An analysis of response times revealed no significant differences between the Tai Chi and non-Tai Chi groups on the More1 (*t*_(43)_ = −0.496, *p* = 0.623), More2 (*t*_(43)_ = −1.190, *p* = 0.240), or More12 tasks (*t*_(43)_ = 0.533, *p* = 0.597), with means for the overall group indicating similar performance across all measures. This pattern of non-significant findings was consistent for male participants, who showed no significant differences on the More1 (*t*_(22)_ = −0.836, *p* = 0.412), More2 (*t*_(22)_ = −1.322, *p* = 0.200), or More12 tasks (*t*_(22)_ = −0.562, *p* = 0.580). For female participants, however, a significant difference was observed on the More12 task, where Tai Chi students exhibited significantly slower reaction times (M = 956.53 ms, SD = 102.06 ms) compared to their non-Tai Chi counterparts (M = 836.87 ms, SD = 86.97 ms), *t*_(19)_ = 2.717, *p* = 0.014. No significant differences were found for females on the More1 (*t*_(19)_ =0.761, *p* = 0.456) or More2 (*t*_(19)_ = 0.678, *p* = 0.506) tasks. These findings suggest that while long-term Tai Chi practice does not affect reaction time performance for males, it may be associated with slower reaction times on a specific task for females. This difference may be attributed to the practitioners' greater inclination for reflective attention during tasks (Beilock and Gray, 2012).

#### 4.2.3 N-back task

**Figure 5:**
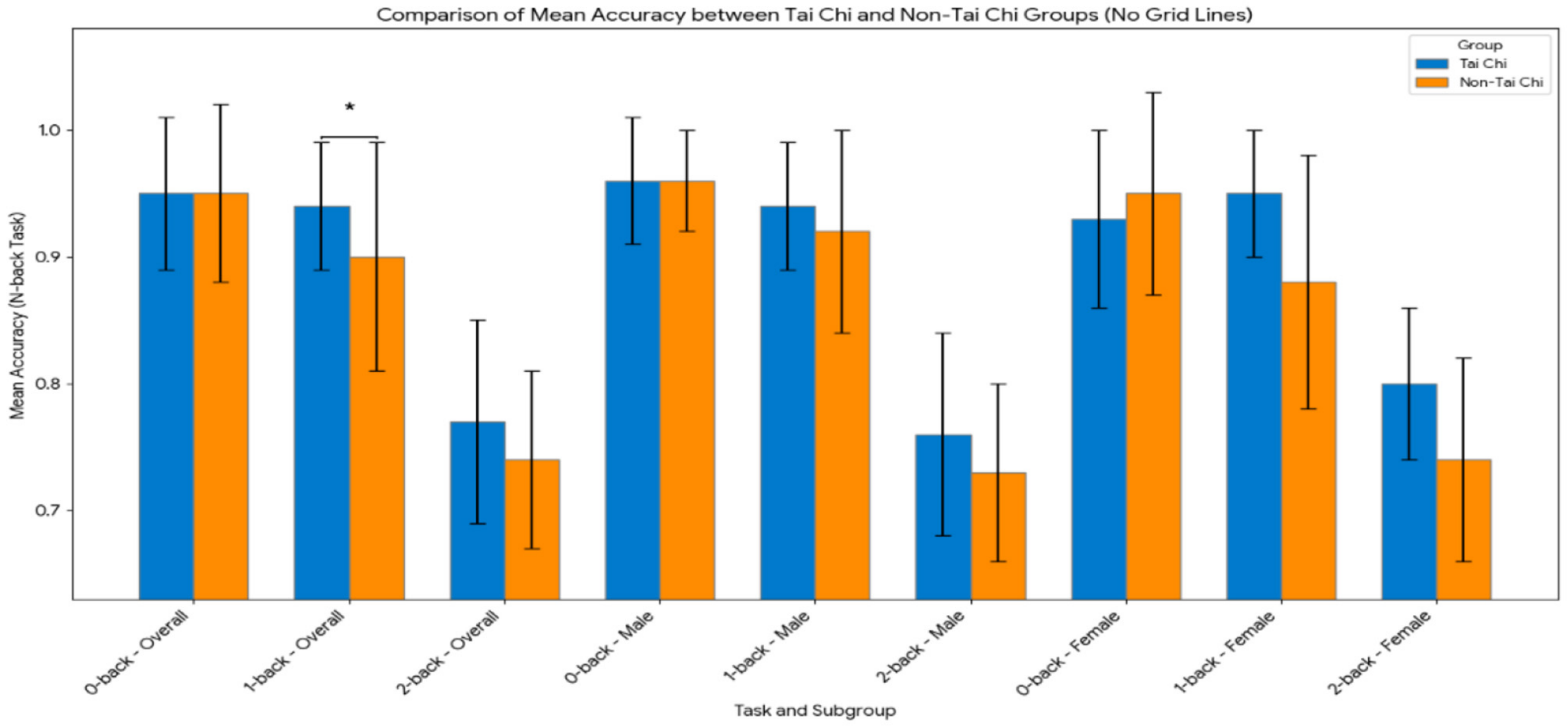
Comparison of mean accuracy rates on the N-back task by group (overall sample). Error bars represent ±1 standard deviation. This figure clearly illustrates the advantage of the Tai Chi group specifically at the 1-back working memory load level. *p < 0.05, **p < 0.001.

N-back is a cognitive training task used to assess and improve working memory. Participants must remember sequences of stimuli (like letters or numbers) and indicate when a current stimulus matches one presented “*n*” steps earlier (Jaeggi et al., 2010). This method targets mental agility and has been linked to enhanced cognitive function and overall brain health. The working memory difference between the two group was assessed through an n-back task comprising of 0-back, 1-back and 2-back task. An independent samples *t*-test was conducted to examine the group and gender differences in performance on three levels of the n-back task between Tai Chi students and non-Tai Chi students as shown on Table 8 and Figure 5.

**Table 8:** N-back task correct responds group difference between the two group.

**Variable**	**Group (mean** ±**std. deviation)**		***t***	***p***	**Male (mean** ±**std. deviation)**		***t***	***p***	**Female (mean** ±**std. deviation)**		***t***	***p***
	**Tai Chi (*****n*** = **21)**	**Non-Tai Chi (*****n*** = **24)**			**Tai Chi (*****n*** = **15)**	**Non-Tai Chi (*****n*** = **9)**			**Tai Chi (*****n*** = **6)**	**Non-Tai Chi (*****n*** = **15)**		
0-back	0.95 ± 0.06	0.95 ± 0.07	0.101	0.92	0.96 ± 0.05	0.96 ± 0.04	0.218	0.83	0.93 ± 0.07	0.95 ± 0.08	−0.507	0.618
1-back	0.94 ± 0.05	0.90 ± 0.09	2.235	0.032^*^	0.94 ± 0.05	0.92 ± 0.08	0.992	0.332	0.95 ± 0.05	0.88 ± 0.10	1.477	0.156
2-back	0.77 ± 0.08	0.74 ± 0.07	1.624	0.112	0.76 ± 0.08	0.73 ± 0.07	1.037	0.311	0.80 ± 0.06	0.74 ± 0.08	1.591	0.128

^*^p < 0.05.

^**^p < 0.01.

The results indicated a significant difference in performance on the 1-back level. Specifically, Tai Chi students (M = 0.94, SD = 0.05) scored significantly higher than non-Tai Chi students (M = 0.90, SD = 0.09), *t*_(43)_ = 2.235, *p* = 0.032. No significant differences were observed in performance on the 0-back level, with Tai Chi students (M = 0.95, SD = 0.06) scoring similarly to non-Tai Chi students (M = 0.95, SD = 0.07), *t*_(43)_ = 0.101, *p* = 0.920. Similarly, no significant difference was observed in performance on the 2-back level, with Tai Chi students (M = 0.77, SD = 0.08) scoring similarly to non-Tai Chi students (M = 0.74, SD = 0.07), *t*_(43)_ = 1.624, *p* = 0.112. These findings suggest that Tai Chi practice may have a positive impact on correct respond at the 1-back level, but does not significantly impact performance on the 0-back or 2-back levels. This result at the 1-back level indicates that Tai Chi student can effective can effectively store and update information in their working memory (Wang et al., 2023).

For males, no significant differences were observed in any of the tasks. Specifically, for the 0-back task, Tai Chi students (M = 0.96, SD = 0.05) whereas to non-Tai Chi students (M = 0.96, SD = 0.04), *t*_(22)_ = 0.218, *p* = 0.830. Similarly, for the 1-back task, Tai Chi students (M = 0.94, SD = 0.05) whereas to non-Tai Chi students (M = 0.92, SD = 0.08), *t*_(22)_ = 0.992, *p* = 0.332. For the 2-back task, Tai Chi students (M = 0.76, SD = 0.08) whereas to non-Tai Chi students (M = 0.73, SD = 0.07), *t*_(22)_ = 1.037, *p* = 0.311.

For females, no significant differences were observed in any of the tasks. Specifically, for the 0-back task, Tai Chi students (M = 0.93, SD = 0.07) whereas to non-Tai Chi students (M = 0.95, SD = 0.08), *t*_(19)_ = −0.507, *p* = 0.618. Similarly, for the 1-back task, Tai Chi students (M = 0.95, SD = 0.05) whereas to non-Tai Chi students (M = 0.88, SD = 0.10), *t*_(19)_ = 1.477, *p* = 0.156. For the 2-back task, Tai Chi students (M = 0.80, SD = 0.06) whereas to non-Tai Chi students (M = 0.74, SD = 0.08), *t*_(19)_ = 1.591, *p* = 0.128. These findings suggest that Tai Chi practice does not significantly impact performance on these specific tasks for either males or females.

In addition to the investigation of differences in correct responses, reaction time differences were also analyzed to assess gender and group disparities between the two groups, For the gender reaction time difference, an independent samples *t*-test was conducted to examine differences in performance on 0-back, 1-back and 2-back tasks between Tai Chi students and non-Tai Chi students as presented in Table 9.

**Table 9:** N-back task reaction time group difference between the two group.

**Variable**	**Group (mean** ±**std. deviation)**		***t***	***p***	**Male (mean** ±**std. deviation)**		***t***	***p***	**Female (mean** ±**std. deviation)**		***t***	***p***
	**Tai Chi (*****n*** = **21)**	**Non-Tai Chi (*****n*** = **24)**			**Tai Chi (*****n*** = **15)**	**Non-Tai Chi (*****n*** = **9)**			**Tai Chi (*****n*** = **6)**	**Non-Tai Chi (*****n*** = **15)**		
0-back	482.36 ± 49.35	484.48 ± 52.78	−0.138	0.891	468.17 ± 41.39	501.26 ± 63.84	−1.548	0.136	517.85 ± 53.20	474.41 ± 44.26	1.922	0.07
1-back	564.92 ± 81.34	565.49 ± 97.69	−0.021	0.983	557.35 ± 71.98	591.54 ± 78.48	−1.09	0.288	583.85 ± 106.57	549.86 ± 107.05	0.658	0.518
2-back	641.93 ± 80.64	632.20 ± 106.92	0.341	0.735	635.94 ± 80.76	658.45 ± 100.48	−0.604	0.552	656.91 ± 85.88	616.45 ± 110.92	0.798	0.435

^*^p < 0.05.

^**^p < 0.01.

The test results revealed no significant differences across any of the tasks. In the 0-back task, Tai Chi students (M = 482.36 ms, SD = 49.35 ms) compared to non-Tai Chi students (M = 484.48 ms, SD = 52.78 ms), *t*_(43)_ = −0.138, *p* = 0.891. Likewise, for the 1-back task, Tai Chi students (M = 564.92 ms, SD = 81.34 ms) compared to non-Tai Chi students (M = 565.49 ms, SD = 97.69 ms), *t*_(43)_ = −0.021, *p* = 0.983. Regarding the 2-back task, Tai Chi students (M = 641.93 ms, SD = 80.64 ms) compared to non-Tai Chi students (M = 632.20 ms, SD = 106.92 ms), *t*_(43)_ = 0.341, *p* = 0.735. These findings suggest that long-term Tai Chi practice does not significantly impact reaction time on these specific tasks.

For males, no significant differences were observed in any of the tasks. Specifically, for the 0-back task, Tai Chi students (M = 468.17 ms, SD = 41.39 ms) whereas to non-Tai Chi students (M = 501.26 ms, SD = 63.84 ms), *t*_(22)_ = −1.548, *p* = 0.136. Similarly, for the 1-back task, Tai Chi students (M = 557.35, SD = 71.98) whereas to non-Tai Chi students (M = 591.54 ms, SD = 78.48 ms), *t*_(22)_ = −1.090, *p* = 0.288. For the 2-back task, Tai Chi students (M = 635.94 ms, SD = 80.76 ms) whereas to non-Tai Chi students (M = 658.45 ms, SD = 100.48 ms), *t*_(22)_ = −0.604, *p* = 0.552.

For females, no significant differences were observed in any of the tasks. Specifically, for the 0-back task, Tai Chi students (M = 517.85 ms, SD = 53.20 ms) whereas to non-Tai Chi students (M = 474.41 ms, SD = 44.26 ms), *t*_(19)_ = 1.922, *p* = 0.070. Similarly, for the 1-back task, Tai Chi students (M = 583.85 ms, SD = 106.57 ms) whereas to non-Tai Chi students (M = 549.86 ms, SD = 107.05 ms), *t*_(19)_ = 0.658, *p* = 0.518. For the 2-back task, Tai Chi students (M = 656.91 ms, SD = 85.88 ms) whereas to non-Tai Chi students (M = 616.45 ms, SD = 110.92 ms), *t*_(19)_ = 0.798, *p* = 0.435. These findings suggest that Tai Chi practice does not significantly impact performance on these specific tasks for either males or females.

The significant difference observed in the 1-back tasks may be attributed to the inherent nature of Tai Chi practice, which emphasizes dual-task performance (Doumas et al., 2009). Tai Chi often necessitates simultaneous movement and mental focus, effectively training the brain to manage multiple tasks concurrently. This engagement enhances working memory, as practitioners must coordinate their physical movements while maintaining cognitive awareness of their surroundings and mental processes (Agmon et al., 2014). The duality of these tasks aligns with the principles demonstrated in the 1-back tasks, where individuals are required to monitor and respond to stimuli while simultaneously recalling previously presented information (Watter et al., 2001).

## 5 Discussion

The results from this study demonstrate a clear, selective impact of long-term Tai Chi practice on executive function. Our findings, drawn from both a self-report measure and objective cognitive tasks, consistently indicate that Tai Chi practitioners exhibit significant advantages in inhibitory control and working memory compared to their non-practitioner counterparts. Specifically, the Tai Chi group had lower BRIEF-A scores on inhibition and working memory, faster reaction times on the Flanker task, and higher accuracy on the 1-back task. Conversely, the study found no significant overall differences in cognitive flexibility, challenging the idea that Tai Chi provides a uniform cognitive enhancement. The research also revealed intriguing gender-specific effects, with male practitioners showing a speed-based advantage in inhibition, while female practitioners demonstrated strategic differences in accuracy and processing time.

### 5.1 Enhancements in inhibition and working memory

The evidence for improved inhibition is robust and consistent across multiple measures. The self-reported BRIEF-A scores indicated that Tai Chi practitioners experienced fewer difficulties with inhibitory control and emotional regulation than the control group (Agmon et al., 2014). This was corroborated by the Flanker task, which showed that the Tai Chi group was significantly faster in responding to both congruent and incongruent stimuli. The lack of a significant accuracy difference in the Flanker task suggests that both groups possessed the fundamental capacity for inhibition. The key distinction lies in the speed and efficiency with which Tai Chi practitioners were able to execute this process, indicating a superior level of attentional regulation and the ability to quickly suppress irrelevant information. This finding is consistent with prior research linking Tai Chi to improved attention and reaction times. Moreover, Tai Chi's rhythmic breathing and meditative qualities contribute to a state of mindfulness that fosters greater self-regulation and a reduction in stress and anxiety (Chen et al., 2021; Hao, 2022). This relaxed, yet focused, mental state is an optimal environment for performing cognitively demanding tasks, which further contributes to enhanced performance (Watter et al., 2001).

Similarly, a significant benefit was observed for working memory. The BRIEF-A results showed that Tai Chi students reported fewer difficulties with working memory, and this was supported by their significantly higher accuracy on the 1-back task. The specificity of this finding is particularly insightful. The 1-back task is the first level of a true working memory challenge, requiring participants to continuously hold and update a single piece of information. Tai Chi's dual-task nature, which necessitates the simultaneous coordination of intricate physical movements and sustained mental focus, directly trains the very cognitive processes required for this level of working memory. The physical movements themselves act as a form of external cueing, while the meditative component enhances the internal monitoring required to track and update mental information. This dual engagement of mind and body creates a unique training environment that effectively strengthens working memory at this specific level of cognitive load. The lack of a significant difference on the more demanding 2-back task suggests that the cognitive benefits of the practice may not extend to more complex, higher-load working memory tasks (Von Gunten et al., 2020).

### 5.2 The paradox of cognitive flexibility

A central and paradoxical finding of this study is the lack of improvement in cognitive flexibility, as reflected by the null findings on the BRIEF-A ‘Shift' subscale and the More-Odd Shift Task. This result contradicts the notion of a uniform cognitive boost and necessitates a deeper explanation. One plausible mechanism for this outcome is the concept of automaticity (Beilock and Gray, 2012), which is a natural consequence of long-term, repetitive practice. The very goal of mastering a Tai Chi form is to make complex movements and sequences effortless and intuitive, thereby reducing the need for conscious cognitive effort. The brain develops automatic reflexes, allowing the practitioner to perform the routine with minimal mental load (Koziol et al., 2012). However, cognitive flexibility, or the ability to switch mental sets, relies on the capacity to break out of established routines and adapt to novel or unanticipated challenges. The mechanism that enhances fluidity in a well-known routine may, therefore, inadvertently suppress the mental agility needed for rapid, adaptive strategy shifts when faced with new stimuli or rules (Taylor-Piliae et al., 2010).

This perspective suggests that the benefits of long-term Tai Chi practice are domain-specific. While the practice excels at training and refining cognitive processes within a structured, repetitive framework; leading to benefits in inhibition and working memory; it may not sufficiently stimulate the novelty-driven processes required for cognitive flexibility (Shen et al., 2021). The paradoxical finding of a significantly slower reaction time on the More-Odd Shift task for female Tai Chi students presents a unique opportunity for interpretation. Instead of representing a cognitive deficit, this slower response may reflect a more deliberate, top-down cognitive process. The enhanced mindfulness and emotional regulation, which the BRIEF-A data suggests for this group, may lead female practitioners to engage in a more controlled, reflective form of attention rather than a quick, reactive shift (Robledo-Castro et al., 2025). This heightened mental control could cause a delay as the practitioner consciously overrides a pre-existing automatic response in favor of a more considered one. This finding is particularly interesting when compared to studies on older adults, where Tai Chi has been shown to improve task-switching performance, which is a form of cognitive flexibility. This age-related difference in outcomes may imply that the benefits of Tai Chi are moderated by the developmental stage of the practitioner. For older adults, the practice may serve a restorative or protective function against age-related cognitive decline, whereas for young adults, the repetitive nature of the practice may not be challenging enough to enhance an already healthy and flexible cognitive system.

While our initial interpretation of the non-significant change in cognitive flexibility focused on the potential persistence of automatic, habitual responses in routine tasks, it is equally important to consider the sensitivity of the task itself given the demographic studied. The More-Odd Shift Task, while effective, may have lacked the necessary challenge to detect subtle differences in cognitive flexibility within a population of young, healthy adults. Performance metrics like accuracy (e.g., More 1 accuracy: 0.95 vs. 0.96) suggest that participants were already performing near a ceiling effect (Wang et al., 2008), making significant improvement or detection of experimental manipulation effects difficult.

### 5.3 The nuance of gender-specific benefits

The study's findings on gender-specific differences suggest that the cognitive benefits of Tai Chi are not uniform but may vary depending on how the practice is engaged. The observation that female Tai Chi students exhibited improved self-reported emotional control and working memory, while male Tai Chi students showed enhanced task initiation and faster reaction times on the Flanker task, points to a potential divergence in the cognitive pathways most affected by the practice. It is possible that male practitioners, on average, focus more on the physical, martial aspects of the form, leading to benefits in reactive control and speed. Several studies involving female groups have demonstrated that Tai Chi training selectively improves emotional memory and enhances emotional regulation ability (e.g., by reducing negative emotional responses or anxiety) through the strengthening of cognitive control mechanisms (Wang et al., 2023; Yuan et al., 2025). Furthermore, meta-analyses suggest that the overall effect size of exercise interventions on executive function may be greater in studies with a higher percentage of female participants (Barha et al., 2017). Conversely, female practitioners may be more attuned to the meditative and emotional regulation components, which could foster improvements in emotional control and a more deliberate, albeit slower, approach to problem-solving. This highlights that the cognitive effects of Tai Chi are not a monolithic benefit but rather a complex interplay of the practice's various components and the individual's mode of engagement.

## 6 Limitations and future directions

A significant limitation of this study is the confounding variable of physical activity level. The Tai Chi group engaged in significantly higher levels of overall physical activity than the control group, making it impossible to definitively attribute the observed cognitive benefits solely to the practice of Tai Chi itself. Future research should address this by employing a more robust study design with a physically active control group that is matched for overall physical activity. An ideal active control group would be matched using Metabolic Equivalent values to better isolate the unique cognitive effects of Tai Chi from the general benefits of exercise.

Secondly, A primary limitation of this study is the small and restricted sample size (n_experimental_=21, n_control_=24). To quantify the potential impact on result reliability, a sensitivity power analysis was conducted for a two-independent-samples *t*-test. Using a conventional significance level of alpha = 0.05 and a desired power of 0.80, the analysis determined that the study was only adequately powered to reliably detect a Minimum Detectable Effect (MDE) of Cohen's d approximate equal to 0.84. This threshold is generally classified as a large effect size (Cohen's guideline: small = 0.2, medium = 0.5, large = 0.8). Consequently, the study was underpowered to detect small or moderate effects that may exist in the population. Any non-significant findings should be interpreted cautiously, as they may represent a Type II error (a false negative) due to insufficient power rather than a true absence of the effect. To address this in future research, expanding recruitment to include non-student young adults and participants from multiple universities is necessary. This will not only improve statistical power but also enhance the breadth of applicability and generalizability of the findings.

A critical methodological limitation of the current study is the failure to account for sex-specific biological factors, particularly the influence of the menstrual cycle on executive function. It is well-established that fluctuating levels of ovarian hormones (e.g., estradiol and progesterone) across the menstrual cycle can significantly modulate performance in cognitive domains such as working memory, inhibitory control, and emotional regulation (Sacher and Villringer, 2013). As our data collection was not controlled for or stratified by cycle phase, this may introduce unaccounted-for biological variability into the cognitive outcome measures for our female participants, potentially obscuring or altering the measured effects of the Tai Chi intervention. Future research involving mixed-sex or exclusively female samples must incorporate either cycle-phase tracking or hormonal assays to minimize this confound and more accurately assess the benefits of long-term Tai Chi practice on executive function.

Thirdly, furthermore, the lack of significant improvement in cognitive flexibility suggests a critical area for future investigation. Subsequent studies should explore whether customized Tai Chi interventions, perhaps by integrating more dynamic, unpredictable sequences or requiring adaptive changes to traditional forms, could mitigate the trade-off of automaticity and enhance cognitive flexibility.

Further research should also delve into the mechanisms driving the observed gender differences. This could be accomplished by employing neuroimaging techniques (such as fMRI) to understand how Tai Chi practice alters brain connectivity and activity in a gender-specific manner. To strengthen the validity and comprehensiveness of future findings, researchers should incorporate objective neurobiological measures like fNIRS or EEG to complement behavioral performance data, and also include informant reports (e.g., from coaches or peers) to provide real-world context for the participants' cognitive and emotional status.

## 7 Conclusion

This study provides compelling evidence that long-term Tai Chi practice offers selective, yet profound, cognitive benefits, notably in enhancing inhibitory control and working memory. The marked improvements observed across both self-report and behavioral measures challenge the notion of a uniform cognitive boost from the practice, highlighting that its benefits are domain-specific. The findings corroborate earlier research that illustrates Tai Chi's capacity to enhance executive functioning through its combination of meditative practices, physical movements, and dual-tasking challenges, which collectively promote neural connectivity and mental discipline. The study's most notable contribution is its illumination of a critical cognitive trade-off: the automaticity fostered by repetitive, long-term practice appears to enhance efficiency in certain domains (inhibition, working memory) while simultaneously limiting improvements in others (cognitive flexibility). The presence of gender-specific findings further enriches this understanding, indicating that the cognitive benefits of Tai Chi are complex and may manifest differently in male and female practitioners. In light of these findings, it is clear that Tai Chi serves as a valuable, holistic strategy for cognitive enhancement, especially for individuals seeking to improve working memory and inhibition. However, for those aiming to enhance cognitive flexibility, supplementary training strategies may be necessary. This study highlights the promise of tailoring Tai Chi protocols to balance the strengths of automaticity with the cognitive demands of novelty, thereby optimizing its potential as a comprehensive tool for cognitive development.

## Data Availability

The raw data supporting the conclusions of this article will be made available by the authors, without undue reservation.
