# Supplementary material for: The selective cognitive benefits of long-term Tai Chi practice on executive function of students: a study on young adults
Source: Front Psychol. 2026 Jan 9;16:1702253. doi: 10.3389/fpsyg.2025.1702253 (PMC12829330; doi:10.3389/fpsyg.2025.1702253)
Supplement: Supplementary file 1 [file Supplementary_file_1.docx]

**Supplementary Information**

| Group | N | Mean | Std. Deviation | t | P |
| --- | --- | --- | --- | --- | --- |
| Tai Chi | 21 | 6460 | 1985 | 11.25 | 0.000** |
| Non-Tai Chi | 24 | 1495 | 414 |  |  |

| * *p*<0.05 ** *p*<0.01 |  |
| --- | --- |

Table S1 IPAQ-S result of the selected student

| Group | Gender | Student | Percent | Mean | Std. Deviation |
| --- | --- | --- | --- | --- | --- |
| Tai Chi (21) | Male | 15 | 63% | 1.29 | 0.463 |
|  | Female | 6 | 37% |  |  |
| Non Tai Chi (24) | Male | 9 | 38% | 1.63 | 0.495 |
|  | Female | 15 | 72% |  |  |

Table S2 demographic distribution of the participants

| Cronbach's Alpha | N of Items |
| --- | --- |
| .971 | 75 |

Table S3 Reliability test analysis of BRIEF-A

|  | | | | |  | | | |
| --- | --- | --- | --- | --- | --- | --- | --- | --- |
| Variable | Tai Chi (Mean±Std. Deviation) | | t | *p* | Non-Tai Chi (Mean±Std. Deviation) | | t | p |
|  | Male (n=15) | Female(*n*=6) |  |  | Male (n=9) | Female (n=15) |  |  |
| Inhibition | 11.40±3.18 | 11.83±3.06 | -0.285 | 0.779 | 12.33±2.69 | 13.93±2.71 | -1.403 | 0.175 |
| Shift | 8.67±2.47 | 10.50±2.51 | -1.531 | 0.142 | 9.56±1.81 | 10.93±1.98 | -1.701 | 0.103 |
| Emotional Control | 12.80±3.12 | 13.83±2.93 | -0.696 | 0.495 | 14.67±4.53 | 18.67±4.05 | -2.244 | 0.035* |
| Self Monitor | 8.67±2.13 | 9.00±2.45 | -0.311 | 0.759 | 9.78±2.54 | 10.13±2.17 | -0.365 | 0.718 |
| Initiate | 11.47±2.50 | 12.33±3.08 | -0.673 | 0.509 | 14.67±2.69 | 15.00±2.85 | -0.283 | 0.780 |
| Working Memory | 11.67±2.89 | 12.17±3.31 | -0.344 | 0.735 | 13.22±3.38 | 15.53±2.92 | -1.769 | 0.091 |
| Plan organize | 14.27±3.15 | 15.17±3.66 | -0.566 | 0.578 | 16.11±4.17 | 17.13±3.31 | -0.665 | 0.513 |
| Task Monitor | 8.73±1.79 | 9.33±2.07 | -0.665 | 0.514 | 10.22±2.05 | 10.53±2.17 | -0.347 | 0.732 |
| Organization of Materials | 10.93±2.37 | 12.33±1.97 | -1.274 | 0.218 | 12.89±2.15 | 13.67±2.74 | -0.726 | 0.476 |
| BRI | 41.53±9.62 | 45.17±10.50 | -0.763 | 0.455 | 46.33±9.42 | 53.67±9.31 | -1.860 | 0.076 |
| MI | 57.07±11.52 | 61.33±13.41 | -0.733 | 0.472 | 67.11±12.22 | 71.87±12.38 | -0.915 | 0.370 |
| GEC | 98.60±20.40 | 106.50±23.60 | -0.768 | 0.452 | 113.44±19.29 | 125.53±20.05 | -1.450 | 0.161 |
| * *p*<0.05 ** *p*<0.01 | | | | |  | | | |

Table S4 The independent samples *t*-test for gender difference within each group

|  | | | | |  | | | |
| --- | --- | --- | --- | --- | --- | --- | --- | --- |
| Variable | Male (Mean±Std. Deviation) | | *t* | *p* | Female (Mean±Std. Deviation) | | *t* | *p* |
|  | Tai Chi (*n*=15) | Non-Tai Chi (*n*=9) |  |  | Tai Chi (*n*=6) | Non-Tai Chi (*n*=15) |  |  |
| Inhibition | 11.40±3.18 | 12.33±2.69 | -0.735 | 0.470 | 11.83±3.06 | 13.93±2.71 | -1.548 | 0.138 |
| Shift | 8.67±2.47 | 9.56±1.81 | -0.936 | 0.359 | 10.50±2.51 | 10.93±1.98 | -0.421 | 0.679 |
| Emotional Control | 12.80±3.12 | 14.67±4.53 | -1.091 | 0.296 | 13.83±2.93 | 18.67±4.05 | -2.644 | 0.016* |
| Self Monitor | 8.67±2.13 | 9.78±2.54 | -1.153 | 0.261 | 9.00±2.45 | 10.13±2.17 | -1.045 | 0.309 |
| Initiate | 11.47±2.50 | 14.67±2.69 | -2.949 | 0.007** | 12.33±3.08 | 15.00±2.85 | -1.895 | 0.073 |
| Working Memory | 11.67±2.89 | 13.22±3.38 | -1.197 | 0.244 | 12.17±3.31 | 15.53±2.92 | -2.299 | 0.033* |
| Plan organize | 14.27±3.15 | 16.11±4.17 | -1.231 | 0.231 | 15.17±3.66 | 17.13±3.31 | -1.195 | 0.247 |
| Task Monitor | 8.73±1.79 | 10.22±2.05 | -1.870 | 0.075 | 9.33±2.07 | 10.53±2.17 | -1.160 | 0.260 |
| Organization of Materials | 10.93±2.37 | 12.89±2.15 | -2.021 | 0.056 | 12.33±1.97 | 13.67±2.74 | -1.078 | 0.295 |
| BRI | 41.53±9.62 | 46.33±9.42 | -1.192 | 0.246 | 45.17±10.50 | 53.67±9.31 | -1.826 | 0.084 |
| MI | 57.07±11.52 | 67.11±12.22 | -2.022 | 0.056 | 61.33±13.41 | 71.87±12.38 | -1.722 | 0.101 |
| GEC | 98.60±20.40 | 113.44±19.29 | -1.760 | 0.092 | 106.50±23.60 | 125.53±20.05 | -1.872 | 0.077 |
| * *p*<0.05 ** *p*<0.01 | | | | |  | | | |

Table S5 The gender difference in executive function between the two group

|  | | | | |  | | | |
| --- | --- | --- | --- | --- | --- | --- | --- | --- |
| Variable | Male Mean±Std. Deviation) | | *t* | *p* | Female (Mean±Std. Deviation) | | *t* | *p* |
|  | Tai Chi (*n*=15) | Non-Tai Chi (*n*=9) |  |  | Tai Chi (*n*=6) | Non-Tai Chi (*n*=15) |  |  |
| Congruence | 0.98±0.02 | 0.98±0.03 | 0.089 | 0.930 | 0.99±0.01 | 0.98±0.04 | 0.640 | 0.530 |
| Incongruence | 0.80±0.11 | 0.86±0.07 | -1.442 | 0.163 | 0.90±0.04 | 0.79±0.16 | 2.344 | 0.031* |
| * *p*<0.05 ** *p*<0.01 | | | | |  | | | |

Table S6 The correct respond gender difference in Flanker task between the two group

|  | | | | |
| --- | --- | --- | --- | --- |
| Variable | Group (Mean±Std. Deviation) | | *t* | *p* |
|  | Tai Chi (*n*=21) | Non-Tai Chi (*n*=24) |  |  |
| Congruence | 0.98±0.02 | 0.98±0.03 | 0.240 | 0.812 |
| Incongruence | 0.83±0.11 | 0.82±0.14 | 0.283 | 0.779 |
| * *p*<0.05 ** *p*<0.01 | | | | |

Table S7 The Flanker task correct responds difference between the two group

|  | | | | |  | | | |
| --- | --- | --- | --- | --- | --- | --- | --- | --- |
| Variable | Male (Mean±Std. Deviation) | | *t* | *p* | Female (Mean±Std. Deviation) | | *t* | *p* |
|  | Tai Chi (*n*=15) | Non-Tai Chi (*n*=9) |  |  | Tai Chi (*n*=6) | Non-Tai Chi (*n*=15) |  |  |
| More1 | 0.93±0.13 | 0.95±0.05 | -0.390 | 0.700 | 0.98±0.03 | 0.97±0.03 | 1.346 | 0.194 |
| More2 | 0.94±0.04 | 0.95±0.03 | -0.253 | 0.803 | 0.95±0.04 | 0.92±0.07 | 0.993 | 0.333 |
| More12 | 0.91±0.05 | 0.91±0.04 | -0.215 | 0.832 | 0.91±0.04 | 0.90±0.09 | 0.338 | 0.739 |
| * *p*<0.05 ** *p*<0.01 | | | | |  | | | |

Table S8 More-Odd Shift Task correct responds gender difference between the two group

|  | | | | |
| --- | --- | --- | --- | --- |
| Variable | Group (Mean±Std. Deviation) | | *t* | *p* |
|  | Tai Chi (*n*=21) | Non-Tai Chi (*n*=24) |  |  |
| More1 | 0.95±0.11 | 0.96±0.04 | -0.529 | 0.600 |
| More2 | 0.95±0.04 | 0.93±0.06 | 0.923 | 0.361 |
| More12 | 0.91±0.05 | 0.90±0.08 | 0.364 | 0.718 |
| * *p*<0.05 ** *p*<0.01 | | | | |

Table S9 More-Odd Shift Task correct responds group difference between the two group

| Independent *t* test | | | | |  | | | |
| --- | --- | --- | --- | --- | --- | --- | --- | --- |
| Variable | Male (Mean±Std. Deviation) | | *t* | *p* | Female (Mean±Std. Deviation) | | *t* | *p* |
|  | Tai Chi (*n*=15) | Non-Tai Chi (*n*=9) |  |  | Tai Chi (*n*=6) | Non-Tai Chi (*n*=15) |  |  |
| 0-back | 468.17±41.39 | 501.26±63.84 | -1.548 | 0.136 | 517.85±53.20 | 474.41±44.26 | 1.922 | 0.070 |
| 1-back | 557.35±71.98 | 591.54±78.48 | -1.090 | 0.288 | 583.85±106.57 | 549.86±107.05 | 0.658 | 0.518 |
| 2-back | 635.94±80.76 | 658.45±100.48 | -0.604 | 0.552 | 656.91±85.88 | 616.45±110.92 | 0.798 | 0.435 |
| * *p*<0.05 ** *p*<0.01 | | | | |  | | | |

Table S10 N-back task reaction time gender difference between the two group

| Independent *t* test | | | | |
| --- | --- | --- | --- | --- |
| Variable | Group (Mean±Std. Deviation) | | *t* | *p* |
|  | Tai Chi (*n*=21) | Non-Tai Chi (*n*=24) |  |  |
| 0-back | 482.36±49.35 | 484.48±52.78 | -0.138 | 0.891 |
| 1-back | 564.92±81.34 | 565.49±97.69 | -0.021 | 0.983 |
| 2-back | 641.93±80.64 | 632.20±106.92 | 0.341 | 0.735 |
| * *p*<0.05 ** *p*<0.01 | | | | |

Table S11 N-back task correct responds group difference between the two group

**Executive Task experiment**

**Flanker task**

The Flanker task consisted of two conditions: congruent and incongruent. In the congruent condition, participants saw a continuous sequence of arrows, either "<<<<<" or ">>>>>", while in the incongruent condition, the sequences "<<><<" or ">><>>" were presented. The formal task comprised four blocks, each containing 30 trials—15 congruent and 15 incongruent. Stimuli were randomly displayed in the center of the screen. Each trial began with a target marker "+" appearing in the center, followed by the stimulus sequence. Afterward, the screen turned black, marking the response time window for participants, who had to respond within 1000 ms. Following their response window, the target marker "+" reappeared, and the cycle restarted. Throughout the task, the screen background was black and the stimuli were white. A 30-second break occurred between blocks, during which participants were instructed to remain quiet and maintain a stable posture. The entire test lasted approximately 5 minutes. During the task, participants had to quickly and accurately identify the direction of the central target arrow among five arrows in the stimulus. The key responses corresponded to the target arrow direction: left "＜" was linked to the "F" key on the keyboard and right "＞" to the "J" key. After each response, the computer recorded the participants' accuracy and reaction time.

**More-odd Shifting Task**

In a study designed to examine cognitive processing, participants were presented with a series of numbers (1-9, excluding 5) displayed centrally on a computer screen. Each number appeared for 2 seconds, followed by a 3-second interstimulus interval. The experiment comprised three distinct tasks. The first task involved no cognitive switching, where participants were instructed to classify numbers as large or small. There were 32 total stimuli. When the number was white, participants pressed the "F" key for values less than 5 and the "J" key for values greater than or equal to 5. In the second task, also containing 16 stimuli, participants judged whether numbers were odd or even. If the number appeared in green, they pressed "F" for odd numbers and "J" for even numbers. The third task introduced cognitive switching, consisting of 64 stimuli, requiring participants to alternate their responses. If the number was displayed in white, they assessed it as large or small, and if it was green, they determined if it was odd or even. The initial two tasks were straightforward, while the third necessitated cognitive flexibility. Participants were instructed to respond as quickly as possible while maintaining accuracy. To minimize variability in performance levels, they underwent 12 practice trials before the main experiment. Consequently, this ensured that the accuracy rates among participants were statistically similar, eliminating significant differences in cognitive processing capabilities across the group.

**N-back Task**

The N-back task comprised three conditions: 0-back, 1-back, and 2-back, utilizing English letters as stimuli. In the 0-back condition, a letter was randomly presented in each block. Participants were instructed to press the "J" key upon the appearance of the letter "X" and to press the "F" key for other letters. During the 1-back and 2-back conditions, letters (excluding "X") were also randomly presented. In the 0-back condition, participants pressed the "J" key if the current letter matched the previous one; in the 1-back condition, they did so if the current letter matched the one before the previous one, while pressing the "F" key for all other letters. The task was structured in a block format, consisting of four blocks for each condition, totaling 12 blocks arranged randomly. Each block began with an 8-second fixation point (+), followed by 4 seconds of task-specific instructions (e.g., for the 0-back condition: "Is the letter X? Yes, press 'J'; No, press 'F' "). Subsequently, eight trials were presented in sequence, with each trial featuring a 1000-ms letter stimulus followed by a 2000-ms blank screen. Participants were allowed to begin pressing the button as soon as the letter stimulus appeared.
